# Set-Membership Estimation for Switched T-S Fuzzy Systems with MDADT Switching in Tunnel Diode Circuits

**DOI:** 10.3390/mi17040402

**Published:** 2026-03-26

**Authors:** Jianghang Xu, You Li, Chaoxu Guan, Zhenyu Wang, Ruiying Liu

**Affiliations:** 1 College of Mechanical Engineering, Jiaxing University, Jiaxing 314001, China; xujianghang92@gmail.com (J.X.);; 2 Zhejiang Academy of Special Equipment Science, Hangzhou 310020, China; 3 Zhejiang Key Laboratory of Special Equipment Safety Technology, Hangzhou 310027, China

**Keywords:** switched T-S fuzzy system, mode-dependent average dwell-time, set-membership estimation, electric circuit, zonotope

## Abstract

This study focuses on the zonotope-based set-membership estimation issue for switched Takagi–Sugeno (T-S) fuzzy systems with application to tunnel diode circuits. Given the practical importance of tunnel diodes in radio-frequency, microwave, and high-speed electronic systems, we first model the tunnel diode circuit as a switched T-S fuzzy system to characterize its inherent dynamics. To address the state estimation issue, we propose a zonotopic set-membership estimation framework for the system under mode-dependent average dwell-time (MDADT) switching, which enables tighter state bounding while ensuring $H_{\infty }$ robustness. A mode-dependent observer is designed to attenuate the effects of external disturbances and measurement noise, and the stability of the estimation error system is analyzed based on an appropriate Lyapunov function. Numerical simulations are conducted and the corresponding results show that the estimated boundary can accurately encompass the true state of the system, and the volume of the estimated set is reduced by approximately 28.99% compared with the interval observer method, thus demonstrating the effectiveness and potential of the proposed approach.

## 1. Introduction

Switched dynamical systems serve as a powerful modeling paradigm for characterizing the dynamic behaviors of practical engineering systems with inherent switching characteristics, which arise from structural reconfiguration, component switching or external control intervention [1,2,3,4]. This has led to their widespread adoption across fields including power electronics, aerospace control, and communication networks [5,6,7]. A central focus in the analysis and synthesis of such systems lies in characterizing the switching signals, which are generally categorized into autonomous and constrained types. Among the most widely studied constrained switching paradigms, the average dwell time (ADT) switching scheme, which enforces a minimum average interval between consecutive switching events, has been widely studied [8,9], though its reliance on mode-agnostic parameters often introduces conservatism. To mitigate this, researchers have extended ADT to the mode-dependent average dwell time (MDADT) paradigm, allowing dwell time constraints to be tailored to each system mode for less restrictive stability criteria [10,11,12]. In recent years, MDADT has been widely applied to the analysis and control of switched linear/nonlinear systems, with research achievements including finite-time stability analysis [13], adaptive control design [14] and tracking control [15]. In contrast to the rich literature on control synthesis, state estimation for switched systems, especially those with strong nonlinearities, remains relatively scarce, creating a critical research gap for practical systems where direct state measurement is infeasible, such as electric circuit systems with switchable components.

Nonlinearity is an inherent and ubiquitous characteristic of modern engineering systems, driven by the growing complexity of industrial processes and operational environments [16,17,18]. Over recent decades, researchers have developed a range of methodologies to address nonlinear control challenges, among which the Takagi–Sugeno (T-S) fuzzy modeling framework has emerged as a particularly effective tool [19,20,21]. Unlike direct nonlinear control strategies, T-S fuzzy systems decompose a global nonlinear behavior into a weighted combination of local linear subsystems, enabling the direct application of mature linear system theories to solve nonlinear control problems. This advantage has propelled the widespread adoption of T-S fuzzy models in areas such as controller synthesis, state observer design, and fault detection, spurring a rich body of theoretical and applied research. Recently, the concept of switched T-S fuzzy systems has gained significant traction, integrating the T-S fuzzy modeling paradigm with switched system theory [22,23]. This integration gives rise to switched T-S fuzzy systems, which perfectly fit the modeling needs of engineering systems with both switching and nonlinear characteristics. For many electric circuit systems, switched T-S fuzzy models have been proven to be an effective modeling approach [24,25,26,27], yet state estimation research for such systems remains in a preliminary stage.

As a critical technique for reconstructing system states, the interval observer methodology has garnered significant research interest in recent years [28,29,30]. In existing research, it is commonly required that an observer gain *L* exists to ensure the matrix (A−LC) is Metzler, which often proves restrictive in practice. This challenge is further amplified for switched systems, where the multimodal nature of the dynamics complicates observer design; for example, existing work [31] imposes the strict dual condition that $\left(A_{i}-L_{i}C_{i}\right)$ matrices must be both Hurwitz and Metzler across all modes, making feasible gain solutions difficult to obtain. Compared with traditional interval estimation, the zonotopic method can effectively mitigate the wrapping effect in iterative estimation, and obtain more accurate state boundary information by constructing generator matrices [32,33,34]. Despite these advances, robust state estimation for switched T-S fuzzy systems remains an under-explored area, leaving a critical gap between theoretical progress and practical engineering needs.

Tunnel diodes are key components of modern low-power and high-speed electronic systems, and their state estimation is the basis of circuit performance regulation and fault diagnosis. However, tunnel diode circuits exhibit both switching characteristics and strong nonlinearities, and are often affected by external disturbances and measurement noise in practical operation, making their state estimation a challenging problem. Against this background, this paper focuses on the zonotope-based set-membership estimation problem for switched T-S fuzzy systems under MDADT switching, using the tunnel diode circuit as a specific application object for research. By constructing an appropriate mode-dependent Lyapunov function, the stability and $l_{2}$ gain performance of the resulting estimation error dynamic system are analyzed under the MDADT switching property. Furthermore, mode-dependent fuzzy observers are designed to ensure robustness against external disturbances. On the basis of the zonotopic set-membership technique, a novel criterion is established to determine the upper and lower bounds of the unknown system states. In the end, numerical simulations based on the tunnel diode circuit model are provided to illustrate the effectiveness of the proposed zonotopic estimation method. The remainder of this paper is organized as follows: Section 2 formulates the problem and presents the system model. Section 3 develops the main theoretical results for zonotopic state estimation. Section 4 provides numerical simulations to validate the effectiveness of the proposed approach, and Section 5 concludes this paper.

**Notation:** In this article, matrix P>0 means that *P* is positive definite. $P^{-1}/P^{T}$ respectively stand for the inverse and transposition of matrix *P*. Symbol * in a matrix expresses a term which can be inferred by symmetry. diag{·} represents a block diagnal matrix. ⊕ is the Minkowski sum and ⊙ is the linear image. |·| on vectors denotes component-wise. A zonotope Z=〈p,G〉, characterized by its center $p\in R^{a}$ and generator matrix $G\in R^{a\times b}$, is a polytopic set formed by mapping the unit hypercube $B^{b}={\left[-1,+1\right]}^{b}$ linearly through *G*, i.e., $Z=p⊕GB^{b}=\left\{p+Gz:z\in B^{b}\right\}$. For arbitrary zonotopes $Z=\langle p,G\rangle ,Z_{1}=\langle p_{1},G_{1}\rangle ,\;Z_{2}=\langle p_{2},\;G_{2}\rangle$ and matrix *K*, the following relations are met:

$$\begin{array}{c} Z_{1}⊕Z_{2}=\langle p_{1}+p_{2},\left[G_{1},G_{2}\right]\rangle ,\\ K⊙\langle p,G\rangle =\langle Kp,KG\rangle ,\\ \langle p,G\rangle \subseteq \langle p,rs(G)\rangle , \end{array}$$

where $rs\left(G\right)=diag\left\{g_{1},\cdots ,g_{a}\right\}$ and $g_{i}=\sum_{n=1}^{b}\left|G_{i,n}\right|,i=1,\cdots ,a$.

## 2. Model Descriptions and Preliminaries

### 2.1. Tunnel Diode Circuit

**Figure 1:**
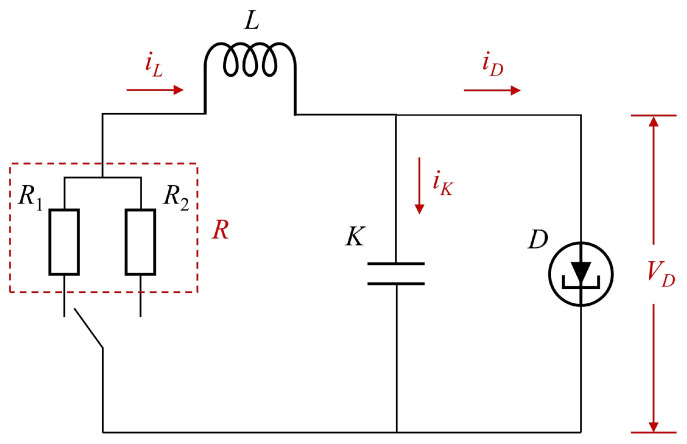
Diagram of the tunnel diode circuit.

In this article, we consider a kind of tunnel diode circuit. By referring to [24,35], the circuit model is shown in Figure 1.

The corresponding mathematical representation of the tunnel diode is expressed as

$$\begin{array}{c} i_{D}\left(t\right)=0.002v_{D}\left(t\right)+0.01v_{D}^{3}\left(t\right). \end{array}$$

**Figure 2:**
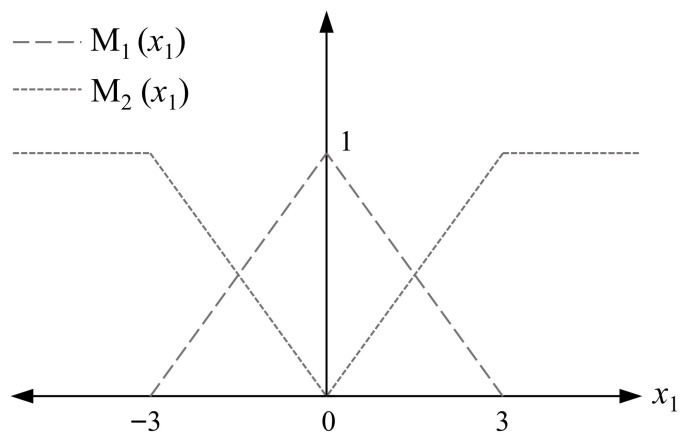
Membership functions for the two fuzzy sets.

Define the state variables as $x_{1}\left(t\right)=v_{D}\left(t\right)$, $x_{2}\left(t\right)=i_{L}\left(t\right)$ and $x\left(t\right)={\left[\begin{array}{cc} x_{1}\left(t\right) & x_{2}\left(t\right) \end{array}\right]}^{T}$. The circuit can be characterized by the following formulas:

$$\begin{array}{cc} {\dot{x}}_{1}\left(t\right)= & -\frac{0.002}{K}x_{1}\left(t\right)-\frac{0.01}{K}x_{1}^{3}\left(t\right)+\frac{1}{K}x_{2}\left(t\right),\\ {\dot{x}}_{2}\left(t\right)= & -\frac{1}{L}x_{1}\left(t\right)-\frac{R_{p}}{L}x_{2}\left(t\right)+\frac{1}{L}\omega \left(t\right),p=1,2,\\ y(t)= & Cx(t)+v(t), \end{array}$$

where y(t), ω(t), and v(t) respectively represent the measurement output, disturbance and noise. *K* and *L* denote the capacitance and inductance. *C* is the sensor matrix. $R_{p}$ is a switchable resistance that takes values between $R_{1}\left(p=1\right)$ and $R_{2}\left(p=2\right)$. The switching rules of the system are determined by the physical operating states of the switchable parallel resistor in the circuit. To handle the nonlinearity, we rewrite the first equation as ${\dot{x}}_{1}\left(t\right)=-\left(\frac{0.002}{K}+\frac{0.01}{K}x_{1}^{2}\left(t\right)\right)\cdot x_{1}\left(t\right)+\frac{1}{K}x_{2}\left(t\right)$ to facilitate sector-based linearization with the constraint $|v_{D}|\;\leq \;3$ by referring to [35]. Local linear models are then derived by evaluating the resulting expression at the operating points $v_{D}=0$ and $v_{D}=\pm 3$, which form the basis of the switched T-S fuzzy model. The fuzzy membership functions $M_{1}\left(x_{1}\right)$ and $M_{2}\left(x_{1}\right)$ are designed as piecewise linear membership functions to match the two local linear models of the tunnel diode, and their construction is determined by the operating voltage constraint and the linearization points. Then, the system can be described as the following switched T-S fuzzy model with the membership functions in Figure 2.

**Plant Rule 1:** IF $x_{1}\left(t\right)$ is $M_{1}\left(x_{1}\right)$ THEN

$$\begin{array}{cc} \dot{x}\left(t\right)= & A_{p1}x\left(t\right)+B_{p1}\omega \left(t\right). \end{array}$$

**Plant Rule 2:** IF $x_{1}\left(t\right)$ is $M_{2}\left(x_{1}\right)$ THEN

$$\begin{array}{cc} \dot{x}\left(t\right)= & A_{p2}x\left(t\right)+B_{p2}\omega \left(t\right). \end{array}$$

where

$$\begin{array}{cc} A_{p1}= & \left[\begin{array}{cc} -\frac{0.002}{K} & \frac{1}{K}\\ -\frac{1}{L} & -\frac{R_{p}}{L} \end{array}\right],\\ A_{p2}= & \left[\begin{array}{cc} -\frac{0.092}{K} & \frac{1}{K}\\ -\frac{1}{L} & -\frac{R_{p}}{L} \end{array}\right],\\ B_{p1}= & B_{p2}={\left[\begin{array}{cc} 0 & \frac{1}{L} \end{array}\right]}^{T},p=1,2. \end{array}$$

From the construction of the above circuit model, the circuit model can be converted to a representation of switched T-S fuzzy systems. To facilitate the subsequent theoretical analysis and digital implementation, the continuous-time tunnel diode circuit model is discretized by the zero-order hold (ZOH) method with the sampling time $T_{s}=0.1$ s. The continuous-time state equations can be converted to the discrete-time form by taking $A_{pi}^{d}=e^{A_{pi}^{c}T_{s}}$ and $B_{pi}^{d}=\left(\int_{0}^{T_{s}}e^{A_{pi}^{c}\tau }d\tau \right)B_{pi}^{c}$, where $A_{pi}^{d},\;B_{pi}^{d}$ stand for the discretized version of system matrices $A_{pi}^{c},\;B_{pi}^{c}$ in the continuous-time scenario.

### 2.2. Switched T-S Fuzzy System Description

Based on the tunnel diode circuit model above, we further generalize it to the discrete-time switched T-S fuzzy system model with switching signal σ(k):[0,∞)→L={1,2,⋯,M}. For σ(k)=p∈L, the *i*-th rule for the *p*-th subsystem is described below. In this case, the tunnel diode circuit is a specific instance of the generalized switched T-S fuzzy system with two switching modes.

**Plant Rule i:** IF $\delta_{p1}\left(k\right)$ is $M_{p1}^{i}$ and ⋯ and $\delta_{ps}\left(k\right)$ is $M_{ps}^{i}$, then

(1) $$\begin{array}{c} \left\{\begin{array}{c} x\left(k+1\right)=A_{pi}x\left(k\right)+B_{pi}\omega \left(k\right),\\ y\left(k\right)=C_{pi}x\left(k\right)+D_{pi}v\left(k\right), \end{array}\right. \end{array}$$

where $x_{k}\in R^{n_{x}},\;y\left(k\right)\in R^{n_{y}},\;\omega \left(k\right)\in R^{n_{\omega }},\;v\left(k\right)\in R^{n_{v}}$. $M_{p1}^{i},\cdots ,\;M_{ps}^{i},\;i\in R=\left\{1,2,\cdots ,r\right\}$ are fuzzy sets and *r* denotes the number of IF-THEN rules. $\delta_{p}\left(k\right)=\left[\delta_{p1}\left(k\right),\cdots ,\delta_{ps}\left(k\right)\right]$ are premise variables. $A_{pi},\;B_{pi},\;C_{pi}$, and $D_{pi}$ are constant real matrices.

By fuzzy blending, the fuzzy model of the *p*-th subsystem is obtained as

(2) $$\begin{array}{c} \left\{\begin{array}{c} x\left(k+1\right)=\sum_{i=1}^{r}\theta_{pi}\left(\delta_{p}\left(k\right)\right)\left[A_{pi}x\left(k\right)+B_{pi}\omega \left(k\right)\right],\\ y\left(k\right)=\sum_{i=1}^{r}\theta_{pi}\left(\delta_{p}\left(k\right)\right)\left[C_{pi}x\left(k\right)+D_{pi}v\left(k\right)\right], \end{array}\right. \end{array}$$

where $\theta_{pi}\left(\delta_{p}\left(k\right)\right)$ are the fuzzy weighting functions satisfying

$$\begin{array}{c} \theta_{pi}\left(\delta_{p}\left(k\right)\right)=\frac{\prod_{n=1}^{s}M_{pn}^{i}\left(\delta_{pn}\left(k\right)\right)}{\sum_{i=1}^{r}\prod_{n=1}^{s}M_{pn}^{i}\left(\delta_{pn}\left(k\right)\right)}\geq 0,\\ \sum_{i=1}^{r}\theta_{pi}\left(\delta_{p}\left(k\right)\right)=1, \end{array}$$

and $M_{pn}^{i}\left(\delta_{pn}\left(k\right)\right)$ is the membership degree of $\delta_{pn}\left(k\right)$ in fuzzy set $M_{pn}^{i}$. In the following text, symbol $\theta_{pi}\left(\delta_{p}\left(k\right)\right)$ is abbreviated as $\theta_{pi}$. Assume that the initial state $x_{0}$, disturbance ω(k) and noise v(k) are unknown but bounded. There exist vectors $x_{0}^{l},x_{0}^{u}\in R^{n_{x}},\;\bar{\omega }\in R^{n_{\omega }},\;\bar{v}\in R^{n_{v}}$ such that

$$\begin{array}{c} x_{0}^{l}\leq x_{0}\leq x_{0}^{u},\\ -\bar{\omega }\leq \omega \left(k\right)\leq \bar{\omega },\\ -\bar{v}\leq v\left(k\right)\leq \bar{v}, \end{array}$$

which yields the zonotope-based representation

(3) $$\begin{array}{c} x_{0}\in \langle {\hat{x}}_{0},G_{0}\rangle ,\omega \left(k\right)\in \langle 0,G_{\omega }\rangle ,v\left(k\right)\in \langle 0,G_{v}\rangle , \end{array}$$

with ${\hat{x}}_{0}=\frac{x_{0}^{u}+x_{0}^{l}}{2},\;G_{0}=diag\left\{\frac{x_{0}^{u}-x_{0}^{l}}{2}\right\},\;G_{\omega }=diag\left\{\bar{\omega }\right\}$, and $G_{v}=diag\left\{\bar{v}\right\}$.

### 2.3. Mode-Dependent Fuzzy Observer

In this study, the premise variable $\delta_{p}\left(k\right)$ of the T-S fuzzy system is directly measurable online via the sensor in the tunnel diode circuit. Since the fuzzy weights $\theta_{pi}\left(\delta_{p}\left(k\right)\right)$ are only functions of the premise variable, the observer can obtain $\theta_{pi}\left(\delta_{p}\left(k\right)\right)$ in real time by calculating the membership functions. This measurable premise variable is a basic assumption for the design of the mode-dependent fuzzy observer, and it is consistent with the actual measurement conditions of the tunnel diode circuit. Then, for the switched T-S fuzzy system (2), the mode-dependent fuzzy observer is constructed as

(4) $$\begin{array}{c} \left\{\begin{array}{c} \hat{x}\left(k+1\right)=\sum_{j=1}^{r}\theta_{pj}\left[A_{pj}\hat{x}\left(k\right)+L_{pj}\left(y\left(k\right)-\hat{y}\left(k\right)\right)\right],\\ \hat{y}\left(k\right)=\sum_{j=1}^{r}\theta_{pj}C_{pj}\hat{x}\left(k\right), \end{array}\right. \end{array}$$

where $\hat{x}\left(k\right)\in R^{n_{x}}$ stands for the observer state and $L_{pj}\left(p\in L,j\in R\right)$ is the gain matrix that remains to be designed. Define $e\left(k\right)=x\left(k\right)-\hat{x}\left(k\right)$ and $d\left(k\right)={\left[\omega^{T}\left(k\right),v^{T}\left(k\right)\right]}^{T}$. From (2) and (4), the observer error system can be derived as

(5) $$\begin{array}{c} e\left(k+1\right)=\underset{i=1}{\sum^{r}}\underset{j=1}{\sum^{r}}\theta_{pi}\theta_{pj}\left[{\bar{A}}_{pij}e\left(k\right)+{\bar{D}}_{pij}d\left(k\right)\right], \end{array}$$

where

$$\begin{array}{cc} {\bar{A}}_{pij}= & A_{pi}-L_{pi}C_{pj},\\ {\bar{D}}_{pij}= & \left[\begin{array}{cc} B_{pi} & -L_{pi}D_{pj} \end{array}\right]. \end{array}$$

Here, we recall the definition of MDADT and a lemma for latter development. For time interval [k,K], let $N_{\sigma p}\left(k,K\right)$ represent the number of activations of the *p*-th subsystem and $H_{p}\left(k,K\right)$ denote its total operating duration. The definition of MDADT is provided below.

**Definition 1**  ([36])**.** *We say that the switching signal σ(k) possesses an MDADT property with parameter $\tau_{ap}$, when positive constant $N_{0p}$ and positive integer $\tau_{ap}\left(p\in L\right)$ exist satisfying*

(6) $$\begin{array}{c} N_{\sigma p}\leq N_{0p}+\frac{H_{p}\left(k,K\right)}{\tau_{ap}},\forall 0\leq k\leq K. \end{array}$$

**Lemma 1**  ([37])**.** *For real-valued matrices M, N and P>0, it follows that*

$$\begin{array}{c} M^{T}PN+N^{T}PM\leq M^{T}PM+N^{T}PN. \end{array}$$

## 3. Zonotopic Set-Membership Estimation

In this article, the main purpose is to develop a zonotopic set-membership estimation approach for the considered circuit model, which integrates the mode-dependent $H_{\infty }$ observer design and a zonotopic estimation algorithm implementation. Under this setup, the estimation problem is formally stated as follows:

**(i)** Determine $H_{\infty }$ observer gain for system (2) such that the error dynamics in (5) is asymptotically stable under MDADT switching and satisfies the $l_{2}$-gain performance, i.e., for scalar γ>0,

(7) $$\begin{array}{c} \underset{k=k_{0}}{\sum^{\infty }}e^{T}\left(k\right)e\left(k\right)\leq \gamma^{2}\underset{k=k_{0}}{\sum^{\infty }}d^{T}\left(k\right)d\left(k\right). \end{array}$$

**(ii)** Establish an algorithm to generate a bounded set containing all system states via using zonotopes.

### 3.1. Stability and $l_{2}$-Gain Analysis

This subsection first focuses on the stability and $l_{2}$-gain analysis of the estimation error system. Theorem 1 establishes sufficient matrix inequality conditions for the asymptotic stability of the error system under MDADT switching signals with a prescribed performance index by employing a mode-dependent Lyapunov function. The introduction of adjustable parameters $\alpha_{p}$ and $\mu_{p}$ in Theorem 1 provides flexible modulation of the decay rate and jump magnitude of the Lyapunov function, laying a rigorous theoretical foundation for the subsequent $H_{\infty }$ observer design.

**Theorem 1.**  *For scalars $0<\alpha_{p}<1,\;\mu_{p}\geq 1,\;p\in L,$ and gamma>0, if there exist matrices $P_{p}>0,\;p\in L$, such that ∀p≠q∈L, ∀i,j∈R,*

(8) $$\begin{array}{c} \left[\begin{array}{cc} -\Psi_{p} & \frac{1}{2}\Psi_{p}\left(\Lambda_{pij}+\Lambda_{pji}\right)\\ {}* & -\Phi_{p} \end{array}\right]<0, \end{array}$$

(9) $$\begin{array}{c} P_{p}-\mu_{p}P_{q}\leq 0, \end{array}$$

*where*

$$\begin{array}{cc} \Psi_{p}= & diag\{P_{p},I\},\\ \Phi_{p}= & diag\{{\bar{\alpha }}_{p}P_{p},\gamma^{2}I\},\\ \Lambda_{pij}= & \left[\begin{array}{cc} {\bar{A}}_{pij} & {\bar{D}}_{pij}\\ I & 0 \end{array}\right], \end{array}$$

*then for a switching signal with the following MDADT property,*

(10) $$\begin{array}{c} \tau_{ap}>\tau_{ap}^{*}=-\frac{ln\mu_{p}}{ln{\bar{\alpha }}_{p}}, \end{array}$$

*the error system (5) is asymptotically stable with a weighted $l_{2}$-gain not larger than $\bar{\gamma }=\gamma \sqrt{\frac{\alpha_{max}}{1-\rho_{max}}\prod_{p=1}^{M}\mu_{p}^{N_{0p}}}$, where ${\bar{\alpha }}_{p}=1-\alpha_{p}$, $\alpha_{max}=max_{p\in L}\left\{\alpha_{p}\right\}$, and $\rho_{max}=max_{p\in L}\left\{\mu_{p}^{\frac{1}{\tau_{ap}}}{\bar{\alpha }}_{p}\right\}$.*

**Proof.**  Choose the Lyapunov function for system (5) as $V_{\sigma (k)}\left(e\left(k\right)\right)=e^{T}\left(k\right)P_{\sigma (k)}e\left(k\right)$. Define $\Pi \left(k\right)=e^{T}\left(k\right)e\left(k\right)-\gamma^{2}d^{T}\left(k\right)d\left(k\right),\;\xi \left(k\right)={\left[e^{T}\left(k\right),d^{T}\left(k\right)\right]}^{T}$. Suppose $k_{s}\left(s\in Z^{+},k_{0}=0\right)$ is the time instant when the system switches and focus on $\sigma \left(k_{s}-1\right)=q\neq \sigma \left(k_{s}\right)=p\in L$. When $k\in [k_{s},k_{s+1})$, we have

$$\begin{array}{cc} \; & V_{p}\left(k+1\right)-{\bar{\alpha }}_{p}V_{p}\left(k\right)+\Pi \left(k\right)\\ = & \underset{i=1}{\sum^{r}}\underset{j=1}{\sum^{r}}\underset{m=1}{\sum^{r}}\underset{n=1}{\sum^{r}}\theta_{pi}\theta_{pj}\theta_{pm}\theta_{pn}\xi^{T}\left(k\right)\left(\Lambda_{pij}^{T}\Psi_{p}\Lambda_{pmn}-\Phi_{p}\right)\xi \left(k\right)\\ = & \frac{1}{4}\underset{i=1}{\sum^{r}}\underset{j=1}{\sum^{r}}\underset{m=1}{\sum^{r}}\underset{n=1}{\sum^{r}}\theta_{pi}\theta_{pj}\theta_{pm}\theta_{pn}\xi^{T}\left(k\right)\left[{\left(\Lambda_{pij}+\Lambda_{pji}\right)}^{T}\Psi_{p}\left(\Lambda_{pmn}+\Lambda_{pnm}\right)-4\Phi_{p}\right]\xi \left(k\right)\\ = & \frac{1}{8}\underset{i=1}{\sum^{r}}\underset{j=1}{\sum^{r}}\underset{m=1}{\sum^{r}}\underset{n=1}{\sum^{r}}\theta_{pi}\theta_{pj}\theta_{pm}\theta_{pn}\xi^{T}\left(k\right)[{\left(\Lambda_{pij}+\Lambda_{pji}\right)}^{T}\Psi_{p}\left(\Lambda_{pmn}+\Lambda_{pnm}\right)\\ \; & +{\left(\Lambda_{pmn}+\Lambda_{pnm}\right)}^{T}\Psi_{p}\left(\Lambda_{pij}+\Lambda_{pji}\right)-8\Phi_{p}]\xi \left(k\right). \end{array}$$

□

By virtue of Lemma 1, it follows that

$$\begin{array}{cc} \; & V_{p}\left(k+1\right)-{\bar{\alpha }}_{p}V_{p}\left(k\right)+\Pi \left(k\right)\\ \leq & \frac{1}{8}\underset{i=1}{\sum^{r}}\underset{j=1}{\sum^{r}}\underset{m=1}{\sum^{r}}\underset{n=1}{\sum^{r}}\theta_{pi}\theta_{pj}\theta_{pm}\theta_{pn}\xi^{T}\left(k\right)[{\left(\Lambda_{pij}+\Lambda_{pji}\right)}^{T}\Psi_{p}\left(\Lambda_{pij}+\Lambda_{pji}\right)\\ \; & +{\left(\Lambda_{pmn}+\Lambda_{pnm}\right)}^{T}\Psi_{p}\left(\Lambda_{pmn}+\Lambda_{pnm}\right)-8\Phi_{p}]\xi \left(k\right)\\ = & \frac{1}{4}\underset{i=1}{\sum^{r}}\underset{j=1}{\sum^{r}}\theta_{pi}\theta_{pj}\xi^{T}\left(k\right)\left[{\left(\Lambda_{pij}+\Lambda_{pji}\right)}^{T}\Psi_{p}\left(\Lambda_{pij}+\Lambda_{pji}\right)-4\Phi_{p}\right]\xi \left(k\right). \end{array}$$

Clearly, $\frac{1}{4}{\left(\Lambda_{pij}+\Lambda_{pji}\right)}^{T}\Psi_{p}\left(\Lambda_{pij}+\Lambda_{pji}\right)-\Phi_{p}<0$ equals condition (8) based on the Schur complement property, which thus means that when $k\in [k_{s},k_{s+1})$,

(11) $$\begin{array}{c} V_{\sigma (k_{s})}\left(k+1\right)\leq {\bar{\alpha }}_{\sigma (k_{s})}V_{\sigma (k_{s})}\left(k\right)-\Pi \left(k\right). \end{array}$$

For the switching instant $k_{s}$, it can be implied from (9) that

(12) $$\begin{array}{c} V_{\sigma (k_{s})}\left(k_{s}\right)\leq \mu_{\sigma (k_{s})}V_{\sigma (k_{s}-1)}\left(k_{s}\right). \end{array}$$

By utilizing inequalities (11) and (12), the stability and $l_{2}$-gain performance can be proven below. First, we consider the asymptotic stability for system (5) with d(k)=0. For any $k\in [k_{s},k_{s+1})$,

$$\begin{array}{cc} V_{\sigma (k_{s})}\left(k\right)\leq & \mu_{\sigma (k_{s})}{\bar{\alpha }}_{\sigma (k_{s})}^{k-k_{s}}V_{\sigma (k_{s-1})}\left(k_{s}\right)\\ \leq & \cdots \\ \leq & \underset{p=1}{\prod^{M}}{\bar{\alpha }}_{p}^{H_{p}\left(k_{0},k\right)}\mu_{p}^{N_{\sigma p}\left(k_{0},k\right)}V_{\sigma (k_{0})}\left(k_{0}\right)\\ \leq & \underset{p=1}{\prod^{M}}\mu_{p}^{N_{0p}}\cdot {\left({\bar{\alpha }}_{p}\mu_{p}^{\frac{1}{\tau_{ap}}}\right)}^{H_{p}\left(k_{0},k\right)}V_{\sigma (k_{0})}\left(k_{0}\right). \end{array}$$

Then, $V_{\sigma (k)}\left(k\right)\to 0$ as time k→∞ when the system switches under MDADT (10) and the asymptotic stability is derived.

In the following, we focus on the weighted $l_{2}$-gain performance for system (5) under zero initial conditions. For any $k\in [k_{s},k_{s+1})$,

$$\begin{array}{cc} V_{\sigma (k_{s})}\left(k\right)\leq & \mu_{\sigma (k_{s})}{\bar{\alpha }}_{\sigma (k_{s})}^{k-k_{s}}V_{\sigma (k_{s-1})}\left(k_{s}\right)-\underset{n=k_{s}}{\sum^{k-1}}{\bar{\alpha }}_{\sigma (k_{s})}^{k-1-n}\Pi \left(n\right)\\ \leq & \cdots \\ \leq & \mu_{\sigma (k_{s})}\cdots \mu_{\sigma (k_{0})}{\bar{\alpha }}_{\sigma (k_{s})}^{k-k_{s}}\cdots {\bar{\alpha }}_{\sigma (k_{0})}^{k_{1}-k_{0}}V\left(0\right)\\ \; & -\mu_{\sigma (k_{s})}\cdots \mu_{\sigma (k_{1})}{\bar{\alpha }}_{\sigma (k_{s})}^{k-k_{s}}\cdots {\bar{\alpha }}_{\sigma (k_{1})}^{k_{2}-k_{1}}\underset{n=k_{0}}{\sum^{k_{1}-1}}{\bar{\alpha }}_{\sigma (k_{0})}^{k_{1}-1-n}\Pi \left(n\right)\\ \; & -\cdots -\underset{n=k_{s}}{\sum^{k-1}}{\bar{\alpha }}_{\sigma (k_{s})}^{k-1-n}\Pi \left(n\right). \end{array}$$

Since $P_{\sigma (k)}>0$ and V(0)=0, it holds that

$$\begin{array}{cc} \; & \underset{n=k_{0}}{\sum^{k-1}}\underset{p=1}{\prod^{M}}\mu_{p}^{N_{\sigma p}\left(n,k\right)}{\bar{\alpha }}_{p}^{H_{p}\left(n,k\right)}e^{T}\left(n\right)e\left(n\right)\\ \leq & \underset{n=k_{0}}{\sum^{k-1}}\underset{p=1}{\prod^{M}}\mu_{p}^{N_{\sigma p}\left(n,k\right)}{\bar{\alpha }}_{p}^{H_{p}\left(n,k\right)}\gamma^{2}d^{T}\left(n\right)d\left(n\right). \end{array}$$

According to $0<\alpha_{p}<1,\;\mu_{p}\geq 1$, we have

$$\begin{array}{cc} \; & \underset{n=k_{0}}{\sum^{k-1}}{\bar{\alpha }}_{min}^{k-1-n}e^{T}\left(n\right)e\left(n\right)\\ \leq & \gamma^{2}\underset{n=k_{0}}{\sum^{k-1}}\underset{p=1}{\prod^{M}}\mu_{p}^{N_{0p}+\frac{H_{p}\left(n,k\right)}{\tau_{ap}}}{\bar{\alpha }}_{p}^{H_{p}\left(n,k\right)}d^{T}\left(n\right)d\left(n\right)\\ \leq & \left(\underset{p=1}{\prod^{M}}\mu_{p}^{N_{0p}}\cdot \gamma^{2}\right)\underset{n=k_{0}}{\sum^{k-1}}\underset{p=1}{\prod^{M}}{\left(\mu_{p}^{\frac{1}{\tau_{ap}}}{\bar{\alpha }}_{p}\right)}^{H_{p}\left(n,k\right)}d^{T}\left(n\right)d\left(n\right)\\ \leq & \left(\underset{p=1}{\prod^{M}}\mu_{p}^{N_{0p}}\cdot \gamma^{2}\right)\underset{n=k_{0}}{\sum^{k-1}}\rho_{max}^{k-1-n}d^{T}\left(n\right)d\left(n\right), \end{array}$$

where ${\bar{\alpha }}_{min}=min_{p\in L}\left\{{\bar{\alpha }}_{p}\right\}=1-\alpha_{max}$. The property (10) gives rise to $0<\rho_{max}<1$. Taking the limit of the above inequality gives

$$\begin{array}{cc} \; & \underset{k=k_{0}+1}{\sum^{\infty }}\underset{n=k_{0}}{\sum^{k-1}}{\bar{\alpha }}_{min}^{k-1-n}e^{T}\left(n\right)e\left(n\right)\\ \leq & \left(\underset{p=1}{\prod^{M}}\mu_{p}^{N_{0p}}\cdot \gamma^{2}\right)\underset{k=k_{0}+1}{\sum^{\infty }}\underset{n=k_{0}}{\sum^{k-1}}\rho_{max}^{k-1-n}d^{T}\left(n\right)d\left(n\right), \end{array}$$

which implies

$$\begin{array}{cc} \; & \underset{n=k_{0}}{\sum^{\infty }}\underset{k=k_{0}+1}{\sum^{\infty }}{\bar{\alpha }}_{min}^{k-1-n}e^{T}\left(n\right)e\left(n\right)\\ \leq & \left(\underset{p=1}{\prod^{M}}\mu_{p}^{N_{0p}}\cdot \gamma^{2}\right)\underset{n=k_{0}}{\sum^{\infty }}\underset{k=k_{0}+1}{\sum^{\infty }}\rho_{max}^{k-1-n}d^{T}\left(n\right)d\left(n\right). \end{array}$$

Together with $\sum_{k=n+1}^{\infty }{\bar{\alpha }}_{min}^{k-1-n}=1/\alpha_{max}$ and $\sum_{k=n+1}^{\infty }\rho_{max}^{k-1-n}=1/\left(1-\rho_{max}\right)$, we obtain

$$\begin{array}{c} \underset{n=k_{0}}{\sum^{\infty }}e^{T}\left(n\right)e\left(n\right)\leq {\bar{\gamma }}^{2}\underset{n=k_{0}}{\sum^{\infty }}d^{T}\left(n\right)d\left(n\right). \end{array}$$

Therefore, the error system is asymptotically stable with a weighted $l_{2}$-gain and the conclusion is drawn.

**Remark 1.**  *Two sets of parameters $\alpha_{p},\mu_{p}\left(p\in L\right)$ are included in Theorem 1. Specifically, $\alpha_{p}\in \left(0,1\right)$ characterizes the admissible rate of decay of the multiple Lyapunov function during the active phase of each subsystem. $\mu_{p}\geq 1$ quantifies the variation coefficient in the multiple Lyapunov function at the instants when switching between subsystems occurs. To ensure the asymptotic stability of the error dynamic system, appropriate choices of these parameters are essential.*

### 3.2. $H_{\infty }$ Observer Design

Despite the stability guarantee provided by Theorem 1, the direct application of Theorem 1 is hindered by the coupling between the Lyapunov matrix $P_{p}$ and the observer gain $L_{pi}$. The subsequent step focuses on coupling these variables through appropriate matrix congruence transformations, and designing the mode-dependent $H_{\infty }$ observer gain for system (2) on the basis of the stability and $l_{2}$-gain analysis results.

**Theorem 2.**  *For scalars $0<\alpha_{p}<1,\;\mu_{p}\geq 1,\;p\in L$, and γ>0, if there exist matrices $P_{p}>0,\;R_{p},\;Y_{pi},\;p\in L,\;i\in R$ such that ∀p≠q∈L, ∀i,j∈R, condition (9) holds and*

(13) $$\begin{array}{c} \left[\begin{array}{cc} {\tilde{\Psi }}_{p} & \frac{1}{2}\left({\tilde{\Lambda }}_{pij}+{\tilde{\Lambda }}_{pji}\right)\\ {}* & -\Phi_{p} \end{array}\right]<0, \end{array}$$

*where*

$$\begin{array}{cc} {\tilde{\Psi }}_{p}= & diag\{P_{p}-R_{p}-R_{p}^{T},-I\},\\ {\tilde{\Lambda }}_{pij}= & \left[\begin{array}{cc} {\tilde{A}}_{pij} & {\tilde{D}}_{pij}\\ I & 0 \end{array}\right],\\ {\tilde{A}}_{pij}= & R_{p}A_{pi}-Y_{pi}C_{pj},\\ {\tilde{D}}_{pij}= & \left[\begin{array}{cc} R_{p}B_{pi} & -Y_{pi}D_{pj} \end{array}\right], \end{array}$$

*then for a switching signal with the following MDADT property (10), the error system (5) is asymptotically stable with a weighted $l_{2}$-gain not larger than $\bar{\gamma }$ and $H_{\infty }$ observer gains are obtained by $L_{pi}=R_{p}^{-1}Y_{pi}$.*

**Proof.**  Pre-multiplying and post-multiplying inequality (8) by $diag\{R_{p}P_{p}^{-1},I,I,I\}$ and its transpose yields

(14) $$\begin{array}{c} \left[\begin{array}{cc} {\bar{\Psi }}_{p} & \frac{1}{2}\left({\tilde{\Lambda }}_{pij}+{\tilde{\Lambda }}_{pji}\right)\\ {}* & -\Phi_{p} \end{array}\right]<0, \end{array}$$

where ${\bar{\Psi }}_{p}=diag\left\{-R_{p}P_{p}^{-1}R_{p}^{T},-I\right\}$. Due to $-R_{p}P_{p}^{-1}R_{p}^{T}\leq P_{p}-R_{p}-R_{p}^{T}$, we know that (13) is sufficient in establishing (8). Then, the proof is straightforwardly completed by applying the results in Theorem 1. □

**Remark 2.**  *From condition (13), we can see that the scalar γ reflects the robustness performance of the underlying system. To mitigate the impact of uncertainties and ensure the precision of estimation results, it is crucial to minimize the value of γ. To achieve this goal, we formulate the optimization problem as*

$$\begin{array}{c} \min\;\gamma ,\;s.t.\;(9)\;\mathrm{and}\;(13), \end{array}$$

*whose solution yields the desired $H_{\infty }$ observer.*

### 3.3. Set-Membership Estimation Approach

Building upon the mode-dependent $H_{\infty }$ observer, this subsection formulates a zonotope-based interval estimation approach for the circuit system described in (2) that robustly handles uncertainties induced by external disturbances and measurement noise. For notational convenience, we rewrite the error dynamics in (5) as

(15) $$\begin{array}{c} e\left(k+1\right)={\bar{A}}_{\eta (k)}e\left(k\right)+{\bar{D}}_{\eta (k)}d\left(k\right), \end{array}$$

where

$$\begin{array}{c} |\begin{array}{cc} {\bar{A}}_{\eta (k)} & {\bar{D}}_{\eta (k)} \end{array}|=\underset{i=1}{\sum^{r}}\underset{j=1}{\sum^{r}}\theta_{pi}\theta_{pj}\left|\begin{array}{cc} {\bar{A}}_{pij} & {\bar{D}}_{pij} \end{array}\right|. \end{array}$$

Assuming that the zonotope $\langle {\hat{x}}_{k},G_{k}\rangle$ encloses the state $x_{k}$, according to the definition of $e_{k}$, we have $e_{k}\in \langle 0,G_{k}\rangle$. Since $\omega \left(k\right)\in \langle 0,G_{\omega }\rangle ,v\left(k\right)\in \langle 0,G_{v}\rangle$, it is easy to get $d\left(k\right)\in \langle 0,G_{d}\rangle$ with $G_{d}=diag\left\{G_{\omega },G_{v}\right\}$. From expression (15), it holds that

(16) $$\begin{array}{cc} e_{k+1}\in & {\bar{A}}_{\eta (k)}⊙\langle 0,G_{k}\rangle ⊕{\bar{D}}_{\eta (k)}⊙\langle 0,G_{d}\rangle \\ = & \langle 0,G_{k+1}\rangle , \end{array}$$

where $G_{k+1}=\left[\begin{array}{cc} {\bar{A}}_{\eta (k)}G_{k} & {\bar{D}}_{\eta (k)}G_{d} \end{array}\right]$.

To avoid the significant computational burden, the reduction operation needs to be taken into account to govern the dimension of generator matrix $G_{k+1}$. Denote its reduced matrix as ${\bar{G}}_{k+1}$. By applying the reduction operation from [38], $e_{k+1}\in \langle 0,{\bar{G}}_{k+1}\rangle$ holds. Substituting this into $x_{k+1}={\hat{x}}_{k+1}+e_{k+1}$ readily leads to the inclusion $x_{k+1}\in \langle {\hat{x}}_{k+1},{\bar{G}}_{k+1}\rangle$. The detailed order reduction process can be found in Algorithm 1 of [33]. Based on the deduction above, the following theorem can be derived for the set-membership estimation.

**Theorem 3.**  *Consider the switched T-S fuzzy system (2). For ∀σ(k)=p∈L, if $x_{k}\in \langle {\hat{x}}_{k},G_{k}\rangle$, then $x_{k+1}\in \langle {\hat{x}}_{k+1},{\bar{G}}_{k+1}\rangle$.*

*Then, for the switched T-S fuzzy system (2), the interval estimation with a prescribed ideal order κ can be given by $\left[x_{n,k}^{l},x_{n,k}^{u}\right],n\in \left\{1,2,\cdots ,n_{x}\right\}$, where*

$$\begin{array}{cc} x_{n,k}^{l}= & {\hat{x}}_{n,k}-\underset{m=1}{\sum^{\kappa }}\left|{\bar{G}}_{n,m}\right|,\\ x_{n,k}^{u}= & {\hat{x}}_{n,k}+\underset{m=1}{\sum^{\kappa }}\left|{\bar{G}}_{n,m}\right|. \end{array}$$

The overall workflow of the proposed algorithm can be summarized as follows:

**(a)** Initialization: Construct the initial state zonotope $\langle {\hat{x}}_{0},G_{0}\rangle$ from the given conditions and uncertainty zonotope $\langle 0,G_{d}\rangle$;

**(b)** Observer Update: Compute the state estimate and output estimate using the designed $H_{\infty }$ observer;

**(c)** Zonotope Recursion: Calculate the error zonotope by (16) to capture the uncertainty propagation;

**(d)** Order Reduction: Utilize Algorithm 1 in [33] to obtain the order-reduced generator matrix ${\bar{G}}_{k+1}$;

**(e)** State Enclosure: Update the state zonotope $\langle {\hat{x}}_{k+1},{\bar{G}}_{k+1}\rangle$ via the Minkowski sum, which provides the set-membership estimate of the true state.

## 4. Numerical Results

Referring back to the tunnel diode circuit presented in Section 2.1, we set the circuit parameters as K=20 mF, L=1000 mH, $R_{1}=10\;\Omega$ and $R_{2}=20\;\Omega$. By taking the discretization time step as 0.1 s, the electric circuit can be expressed by switched T-S fuzzy system (1) with two modes. The corresponding system matrices are calculated as

$$\begin{array}{cc} A_{11}= & \left[\begin{array}{cc} 0.8144 & 2.8909\\ -0.0578 & 0.2420 \end{array}\right],A_{12}=\left[\begin{array}{cc} 0.5037 & 2.2417\\ -0.0448 & 0.2616 \end{array}\right],\\ A_{21}= & \left[\begin{array}{cc} 0.8542 & 1.9834\\ -0.0397 & 0.0648 \end{array}\right],A_{22}=\left[\begin{array}{cc} 0.5345 & 1.4842\\ -0.0297 & 0.0774 \end{array}\right],\\ B_{pi}= & {\left[\begin{array}{cc} 1 & 1.1052 \end{array}\right]}^{T},p\in L,i\in R, \end{array}$$

and choose

$$\begin{array}{c} C_{pi}=\left[\begin{array}{cc} 1 & 0 \end{array}\right],D_{pi}=1. \end{array}$$

Taking $\alpha_{1}=0.12,\alpha_{2}=0.10,\mu_{1}=1.10,\mu_{2}=1.15$, and γ=1.75 into account, and the fact that the inequalities in Theorem 2 are solvable and based on the MDADT constraint (10), the MDADT can be calculated as $\tau_{a1}^{*}=0.7456$ and $\tau_{a2}^{*}=1.3265$. The $H_{\infty }$ observer gains are obtained as

$$\begin{array}{cc} L_{11}= & \left[\begin{array}{c} 2.5464\\ 0.5615 \end{array}\right],L_{12}=\left[\begin{array}{c} 1.8270\\ 0.4838 \end{array}\right],\\ L_{21}= & \left[\begin{array}{c} 2.1533\\ 0.4313 \end{array}\right],L_{22}=\left[\begin{array}{c} 1.5090\\ 0.3452 \end{array}\right]. \end{array}$$

**Figure 3:**
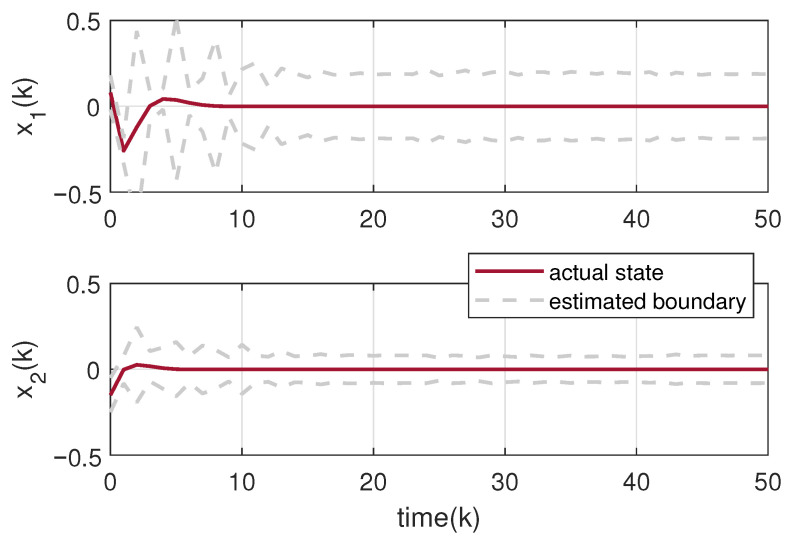
Set-membership estimation of the system state under $x_{0}={\left[0.08,-0.15\right]}^{T}$.

**Figure 4:**
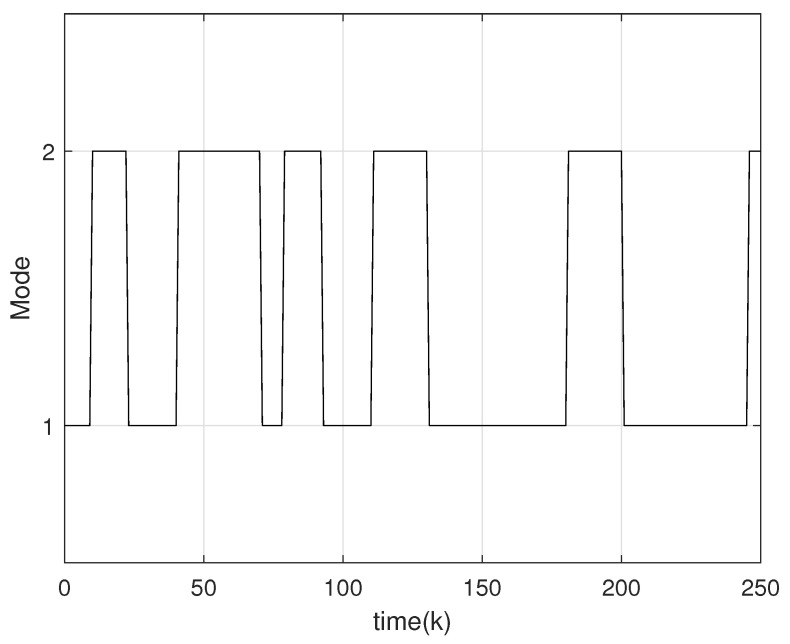
MDADT switching signal.

**Figure 5:**
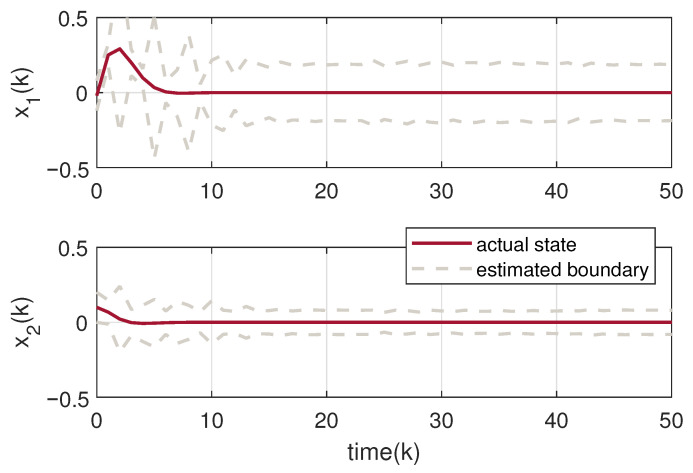
Set-membership estimation of the system state under $x_{0}={\left[-0.02,0.10\right]}^{T}$.

The circuit system is initialized with a state vector $x_{0}={\left[0.08,-0.15\right]}^{T}$. The uncertainties including disturbance and noise are assumed within $\langle 0,G_{d}\rangle$ and $G_{d}=diag\left\{0.1,0.1\right\}$. Based on the designed observer gains, we exploit Theorem 3 to obtain the set-membership estimation of the states of the tunnel diode circuit. The true state trajectory of the system and its estimated zonotopic bounds are drawn in Figure 3 with the switching signal in Figure 4. It can be calculated from Figure 4 that the actual average dwell times of the two subsystems can be calculated as approximately 24.8 and 17, fully satisfying the required MDADT constraint $\tau_{ap}>\tau_{ap}^{*}$ in (10). To further validate the generality of the proposed method, we extend the numerical verification to multiple scenarios by varying the initial conditions and introducing random uncertainties. Figure 5 displays the zonotopic estimation results under initial state $x_{0}={\left[-0.02,0.10\right]}^{T}$ with random bounded disturbance and noise. From these figures, it is evident that the actual state trajectories remain strictly contained within the zonotopic enclosure, thereby confirming the efficacy of the proposed set-membership estimation approach. Furthermore, we compare the proposed zonotopic estimation with the interval observer method in [39]. As shown in Table 1, the interval widths at k=50 are ${\left[\begin{array}{cc} 0.4532 & 0.1827 \end{array}\right]}^{T}$ for our proposed method and ${\left[\begin{array}{cc} 0.5103 & 0.2285 \end{array}\right]}^{T}$ for the interval observer method in [39]. The corresponding set volumes are calculated as 0.0828 and 0.1166, respectively, indicating that the proposed zonotopic method achieves a 28.99% reduction in set volume compared with the interval observer approach, which confirms its lower conservatism in state bounding. In addition, Table 2 presents a comparison of computational time and the root-mean-square value of $rs({\bar{G}}_{k})$ under different ideal orders of reduction operation. It clearly demonstrates that increasing the ideal zonotope order improves state estimation accuracy but prolongs computation. Consequently, selecting a suitable zonotope order requires a deliberate compromise between estimation performance and computational efficiency.

## 5. Conclusions

This paper investigates the zonotope-based set-membership estimation for switched T-S fuzzy systems and its application to tunnel diode circuit state estimation. A switched T-S fuzzy model is first established to characterize the nonlinear and switching dynamics of a circuit model. By constructing a mode-dependent Lyapunov function, the stability and $l_{2}$-gain performance of the estimation error system under MDADT switching are analyzed, and a mode-dependent $H_{\infty }$ observer is designed to enhance robustness against disturbances and noise. A novel zonotopic set-membership criterion is proposed to determine the bounds of unknown states, and numerical simulations on the tunnel diode circuit verify that the proposed method achieves satisfactory estimation accuracy and effective robustness, with actual states strictly enclosed in the derived zonotopic bounds. It should be noted that the present work has certain limitations that merit further exploration, including limited numerical validation and slight conservatism in the adopted zonotope reduction strategy. For future research, the proposed estimation framework can be extended to complex switched T-S fuzzy systems with time delays, stochastic disturbances or parameter uncertainties by adjusting the Lyapunov function and stability criteria. It can also be combined with adaptive control strategies and applied to more practical electric circuit and industrial nonlinear switching systems to further improve its engineering application value.

## Figures and Tables

**Table 1:** Interval estimation widths by different methods (k = 50).

Methods	Interval Widths
$|x_{k}^{u}-x_{k}^{l}|$ by Theorem 3	${\left[\begin{array}{cc} 0.4532 & 0.1827 \end{array}\right]}^{T}$
$|x_{k}^{u}-x_{k}^{l}|$ by [39]	${\left[\begin{array}{cc} 0.5103 & 0.2285 \end{array}\right]}^{T}$

**Table 2:** Comparison results under different ideal orders.

Ideal Order	5	10	15
**Computation time**	0.052	0.071	0.096
$rs({\bar{G}}_{k})$	0.078	0.075	0.073

## Data Availability

All original data of this study is included herein, while additional inquiries can be directed towards the corresponding author.
